# Real-Time Monitoring of NIH/3T3 Cell Growth and Drug Reaction Using Impedance Biosensors and Comparison with Biological Assays

**DOI:** 10.3390/bios15120788

**Published:** 2025-12-01

**Authors:** Seok-kyu Kim, Gayoung Lee, Yeeun Kim, Dahyun Kang, Moongyu Jang

**Affiliations:** 1 School of Nano Convergence Technology, Hallym University, Chuncheon 24252, Republic of Korea; 2 Center of Nano Convergence Technology, Hallym University, Chuncheon 24252, Republic of Korea

**Keywords:** semiconductor, NIH 3T3 cell, puromycin, ECIS, impedance, frequency

## Abstract

Impedance biosensors are manufactured on glass slides using a semiconductor process to monitor cell growth and cell–drug reactions in real time, and the results are compared with biological assay results to confirm the validity of impedance measurement method. Approximately 10,000 cells per well were cultured for 48 h, after which 6.67 μg/mL puromycin was injected to observe apoptosis over the following 48 h. A frequency sweep from 1 kHz to 1 MHz was performed to determine the optimal frequency range, identifying 367–440 kHz as the most sensitive for detecting impedance changes. Impedance was measured every 10 min for 96 h. Capacitance gradually increased during cell proliferation, while after drug administration, a transient increase occurred within 9 h, followed by a rapid decline, indicating cell death within 24 h. The sensor utilized Electrical Cell–substrate Impedance Sensing (ECIS) to detect real-time changes in cell status without the need for staining or destruction. Comparison with conventional biological assays such as MTS and FACS confirmed that the impedance biosensor provided higher sensitivity and quantitative accuracy in monitoring both cell proliferation and apoptosis. This study demonstrates that the developed biosensor enables label-free, non-invasive, and continuous monitoring of cellular behaviors with acceptable coincidence with 3 different biological assay results. Impedance biosensor presents a promising alternative to conventional biological assays and offers potential applications in drug screening, cytotoxicity evaluation, and real-time biological monitoring.

## 1. Introduction

Conventional methods for measuring cell growth and death involve staining cells and observing them directly under a microscope, allowing assessment of cell density and morphology [1,2]. In a study by Giaever and Keese in the late 20th century, cells at various densities were cultured on gold electrodes deposited via evaporation, and external electric fields were applied to measure overall impedance. Their results suggested that the measured impedance reflected changes in cell density and morphology [3]. Following this, researchers introduced the Cell–substrate Impedance Sensor (CIS) to observe cell behavior through electrical signals [4]. In the 1990s, a more advanced system called Electric Cell–Substrate Impedance Sensing (ECIS) was developed [5,6].

Traditional biological assays, MTT and Fluorescence-activated cell sorting (FACS) are widely used. These biological assays are well known and are used as reference methods for cell growth and drug reaction monitoring. However, they cannot provide real-time, continuous observation of living cells. Furthermore, these methods require multiple sample preparations, increasing the likelihood of measurement errors. Due to these limitations, ECIS is gaining attention as a promising alternative for a wide range of biological applications. Compared traditional biological assay, ECIS provides continuous and real-time monitoring of cell behavior.

Compared with our previous impedance biosensor studies, this work newly focuses on the systematic validation of the impedance-based measurements by directly benchmarking them against three standard biological assays (MTS, FACS and Annexin V) under the same experimental conditions. We show that the impedance biosensor provides label-free, continuous monitoring while maintaining acceptable coincidence with conventional assays, and in many cases reveals earlier apoptotic changes that are not captured by endpoint biological methods.

To position our approach within the existing literature, representative impedance-based studies on adherent cell cultures (refs. [7,8,9,10,11]) were surveyed and are summarized in Table 1. These works cover ECIS type systems applied to cell proliferation, cytotoxicity testing, and barrier-function monitoring. While they clearly demonstrate the advantages of label-free, real-time impedance monitoring over conventional endpoint assays, most focus on qualitative impedance changes at a single or proprietary frequency index and rarely include systematic frequency-sweep analysis or direct, quantitative comparison with multiple biological assays. In contrast, the present study combines frequency-optimized impedance measurements with side-by-side validation against three independent biological assays under matched conditions, thereby providing an integrated assessment of the impedance method for cell growth and drug-reaction monitoring.

The ECIS analysis is a method of examining the cell state by observing the change in impedance according to the contact area and contact degree between the cell and the sensor electrode. Measured impedance values are strongly affected with the existence of cells. If there is no cell on the electrode, a current flow without any other interference. However, when cells are placed on the electrode, the cell membrane is a phospholipid double layer insulator, so current flows between the cells [12,13,14]. The current measured through these various paths has a great influence on the measured impedance, so that the shape and condition of the cell can be observed by impedance change. In addition, ECIS methods are convenient and non-invasive by enabling the real-time monitoring of cell conditions with in vitro measurements [8,15,16].

Extensive research and development using the ECIS method are currently underway, with numerous applications documented in recent studies. ECIS systems have been applied across various fields, including cancer research, virology, drug composition analysis, and food safety, as well as in cellular biology for analyzing cell behavior and cytotoxicity [17,18,19,20,21,22,23,24,25]. Additionally, ongoing studies are exploring sensor structure optimization and advanced analysis techniques, contributing to a growing body of literature on real-time cell monitoring [26,27,28].

In this study, the processes of cell growth and apoptosis were observed in real time, demonstrating high accuracy and sensitivity of the developed system. By comparing the electrical signals from the impedance biosensor with conventional biological measurements, we confirmed that the electrical signals accurately reflect biological changes such as cell proliferation and drug responses. An impedance biosensor was fabricated on a glass slide, and the growth of NIH/3T3 cells, as well as apoptosis induced by puromycin, was monitored electrically. These results were compared with a control sample containing only medium to verify that the observed signals originated from cellular activity. Repeated experiments were conducted to confirm the reproducibility of the results. After that, cell proliferation and death were assessed using MTS assays and FACS, allowing for comparative analysis. The findings demonstrated the superior sensitivity and quantification capability of the impedance biosensor relative to conventional biological methods.

## 2. Materials and Methods

### 2.1. Manufacturing Processes of Impedance Biosensors

**Figure 1:**
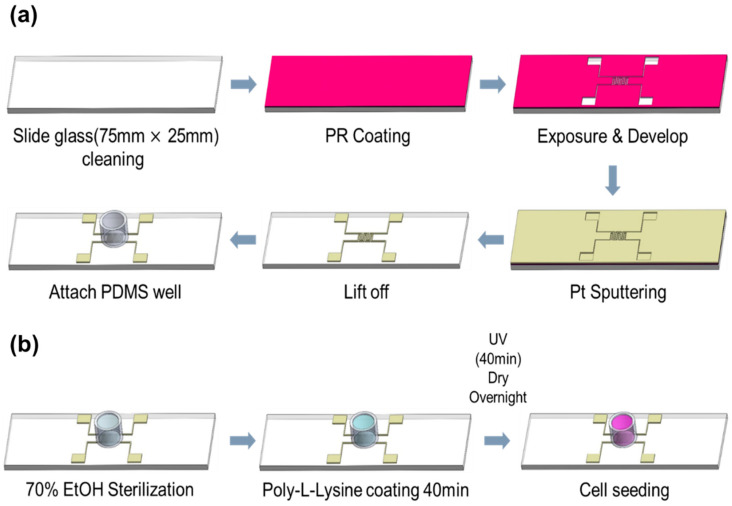
(**a**) Process of manufacturing impedance biosensor (**b**) Environmental setting process for cell growth inside sensor well.

**Figure A1:**
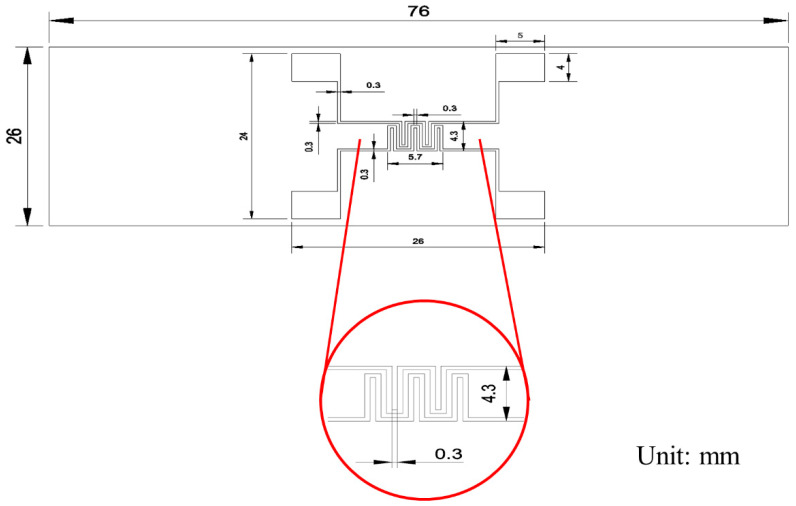
Electrode pattern of impedance biosensor.

Figure 1a illustrates the fabrication process of the impedance biosensor. The biosensors were constructed on transparent slide glass substrates to allow microscopic observation of cell growth and apoptosis. An impedance pattern was manufactured on the slide glass for the monitoring of the variations in impedance with the growth and death of cells, and in this case, platinum (Pt target, e.g., 99.99%, Kojundo Chemical Laboratory Co., Ltd., Sakado, Japan) was used as an electrode to enable the growth of cells on the surface of sensor. The fabrication process involved photolithography for precise patterning. A photoresist (PR, AZ 5214E, Merck Performance Materials GmbH, Darmstadt, Germany) was spin-coated at 4500 RPM for 35 s and soft-baked at 100 °C for 10 min. After aligning the mask, the exposed PR on the electrode region was developed. An 80 nm thick Pt layer was then deposited via sputtering. Prior to Pt deposition, a 5 nm chromium (Cr) adhesion layer was applied to improve bonding between the Pt and SiO_2_ substrate. Following metal deposition, the lift-off process was used to define the electrode pattern. To protect the electrode pads during electrical probing, a 2000 Å thick aluminum (Al) layer was deposited by thermal evaporation. A PDMS well was attached to the electrode area for cell seeding. The PDMS well, fabricated using a biopsy punch, had an inner diameter of 0.8 cm and an outer diameter of 1.0 cm. The complete process is depicted in Figure 1. The final electrode pattern had a line width of 0.3 mm and an inter-electrode spacing of 0.3 mm, as shown in Figure A1.

### 2.2. Creating a Cell Culture Environment Within PDMS Well

To prepare a suitable environment for cell culture, additional surface treatment was conducted on the sensors fabricated as described in Section 2.1, as illustrated in Figure 1b. First, the interior of the PDMS well where cell growth occurs was cleaned and sterilized with 70% ethanol. After that, a 0.01% solution of Poly-L-lysine is coated during 40 min for the attachment of cell, irradiated with ultraviolet (UV) about 40 min for sterilization, and dried overnight. Following this preparation, approximately 10,000 NIH/3T3 cells were seeded into each PDMS well and incubated for 48 h to allow for cell attachment and proliferation.

### 2.3. Principles of Impedance Biosensors

**Figure A2:**
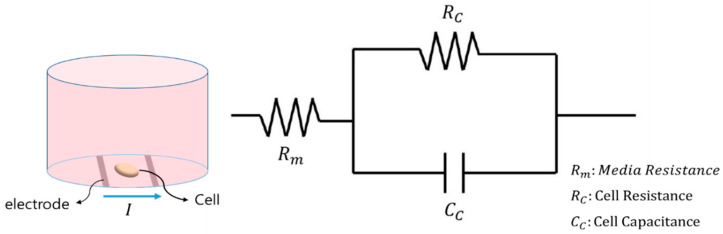
Impedance measurement circuit model for one cell.

As noted in the introduction, impedance biosensors have been continuously developed through the implementation of the Electric Cell–substrate Impedance Sensing (ECIS) system, originating from the foundational work by Giaever and Keese in 1983. The ECIS system enables quantitative analysis of the shape and dynamic behavior of cultured cells. When cells and DMEM are introduced into the well containing the sensor electrodes, the cells settle and adhere to the electrode surface. As cells attach, they interfere with the current flow, leading to changes in the measured impedance [29]. Under the assumption that current is applied to measure individual cells, the system captures both the resistance of the medium and the resistance and capacitance of the cells. The electrical model of this system can be represented as shown in Figure A2.

This research introduces the ECIS system to manufacture an impedance biosensor through the processes of Section 2.1 and Section 2.2 and applies a constant AC between the electrodes. The outer and inner liquids of cells are composed of conductors, and the cell membrane is a phospholipid double layer that acts as an insulator, so electric charges accumulate at the interface between the cell membranes, and each cell acts like a capacitor. Therefore, an increase in the surface area of the cell or the number of cells due to cell growth causes a change in capacitance values [30,31,32,33]. The behavior of the cell can be quantitatively confirmed through a change in the capacitance value measured by the sensor.

**Figure A3:**
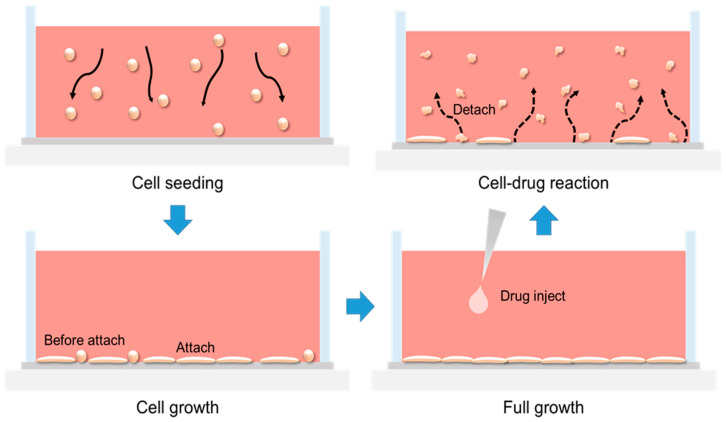
The growth and death process of cells occurring inside the well of the impedance biosensor.

The mechanism occurring inside the impedance biosensor well is illustrated in Figure A3. When cells are seeded, they settle to the bottom of the well by gravity and adhere to the sensor surface. The NIH/3T3 cells used in this study are fibroblasts, which exhibit a round morphology immediately after seeding and gradually spread into a flat, elongated shape upon attachment [34,35]. Once the cells have proliferated and covered the sensor surface, puromycin is introduced to induce apoptosis. Puromycin, a naturally occurring amino nucleoside antibiotic, structurally resembles the 3′ end of aminoacyl-tRNA. During protein synthesis, it incorporates into the elongating peptide chain in place of tRNA, thereby inhibiting ribosomal catalysis and prematurely terminating translation [36,37]. Following puromycin treatment, the cells undergo apoptosis and gradually detach from the electrode surface.

### 2.4. Experimental Conditions and Materials

**Figure A4:**
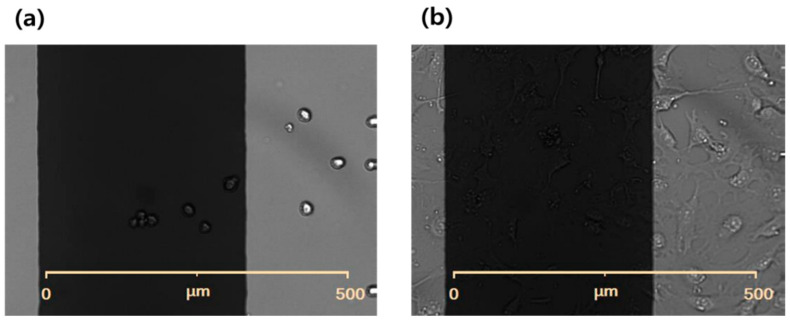
(**a**) Cell growth immediately after NIH/3T3 cell seeding (**b**) 24 h after cell seeding.

The cells used in this study were NIH/3T3 fibroblasts, originally derived from mouse embryonic tissue. These cells typically measure approximately 18 μm immediately after seeding, and can grow to over 100 μm in diameter as they proliferate and adhere to the culture substrate. Figure A4a shows the cells immediately after seeding, while Figure A4b shows their morphology after 24 h of incubation.

For cell culture, Dulbecco’s Modified Eagle Medium (DMEM, Corning, Cat. No. 10-013-CV) containing 4.5 g/L glucose, L-glutamine, and sodium pyruvate was used, supplemented with 10% calf serum and 1% streptomycin. Puromycin (Gibco™, A11138-03, Thermo Fisher Scientific Inc., Waltham, MA, USA) was diluted to a working concentration of 10 μg/mL in culture medium. The CO_2_ incubator was maintained at 37 °C with over 95% humidity and 5% CO_2_ concentration [38].

Under these conditions, cells cultured on the sensor were monitored in real time using both microscopy and electrical measurements. After 48 h, the wells were fully populated with cells. Puromycin was then administered at a concentration of 6.67 μg/mL, and apoptosis was monitored in real time under the same conditions.

**Figure A5:**
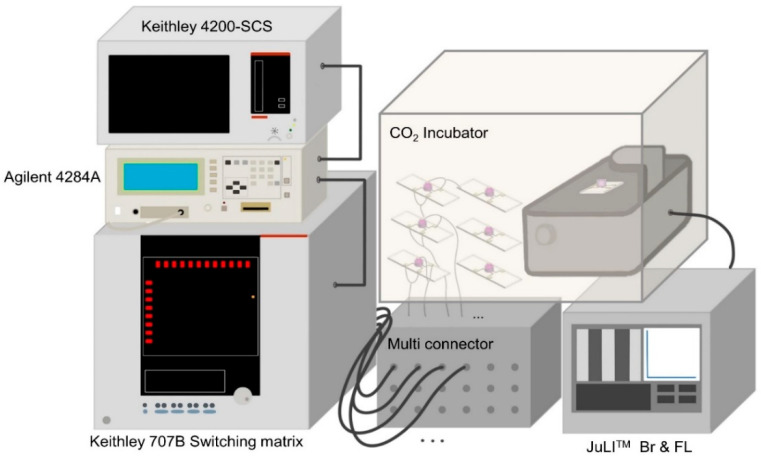
Measurement of cell growth and death with impedance biosensor.

Impedance measurements were conducted using a Keithley 4200-SCS semiconductor (Keithley Instruments, Solon, OH, USA) characterization system connected to an Agilent 4284A LCR meter (Agilent Technologies, Santa Clara, CA, USA) and a Keithley 707B switching matrix (Keithley Instruments, Solon, OH, USA), enabling the sequential measurement of up to six sensors. Real-time microscopic images were captured every 10 min using NanoEnTek’s live-cell imaging system (JuLI™ Br & FL, NanoEnTek Inc., Seoul, Republic of Korea). Figure A5 presents a schematic of the measurement system used to simultaneously monitor cellular growth and death both electrically and optically.

### 2.5. Electrical Measurement Methods

To enhance the reliability of the impedance measurements, the results were compared with those obtained using a conventional biological method capable of assessing cell viability. For this purpose, a cell proliferation assay was conducted to validate cell growth. This assay evaluates the degree of proliferation or viability of living cells based on mitochondrial enzyme activity. Specifically, tetrazolium salts are reduced by mitochondrial enzymes into insoluble purple formazan crystals, and the resulting absorbance correlates with the number of viable cells [39,40,41].

The assay was performed using the CellTiter 96^®^ AQUEOUS One Solution Cell Proliferation Assay (Promega, Madison, WI, USA). NIH/3T3 cells were seeded into five replicate wells at a density of 1 × 10^4^ cells/well and incubated at 37 °C for 4 h to ensure proper attachment. The culture medium was subsequently replaced with MTS reagent at different time points (4, 12, 24, 36, and 48 h after seeding), and cells were incubated for an additional hour. Absorbance was measured at 490 nm using a microplate reader (Thermo Scientific, Waltham, MA, USA). The fold increase in absorbance over time was used to quantify cell proliferation relative to the initial measurement at 4 h.

### 2.6. FACS (Fluorescence-Activated Cell Sorting)

Fluorescence-activated cell sorting (FACS) was employed to assess cell death. FACS is a quantitative method that distinguishes live and dead cells by staining them and analyzing light scattering and fluorescence signals. Specifically, forward scatter (FSC), side scatter (SSC), and fluorescence intensity are measured to evaluate cellular properties and viability. Among various analytical techniques available with FACS, cell cycle analysis was used in this study. This method enables the assessment of all four phases of the cell cycle using flow cytometry. The dead cells are classified as cells that deviate from the cell cycle because the cell cycle stops, and the percentage value of the dead cells among all cells can be confirmed. This approach allowed us to quantitatively monitor drug-induced apoptosis in NIH/3T3 cells after 48 h of culture, providing a complementary validation of the impedance-based measurements [42,43,44].

NIH/3T3 cells were seeded in 6 well plates at a density of 2 × 10^5^ cells/well and treated with puromycin (GibcoTM) at a concentration of 6.67 μg/mL. At various time points between 4 and 45 h post-treatment, cells were harvested using trypsin, washed with phosphate-buffered saline (PBS; Corning, New York, NY, USA), and fixed in 70% ethanol. For DNA staining, RNase (0.2 mg/mL) was added to the cell suspension and incubated for 30 min. Subsequently, Propidium Iodide (PI; 1 μg/mL, BioLegend, San Diego, CA, USA) was added at room temperature for 15 min. Sub-diploid DNA content, indicative of apoptotic cells, was analyzed using flow cytometry with the FACS Calibur system (Becton Dickinson, Franklin Lakes, NJ, USA).

Based on FACS analysis with PI staining, cell cycle distribution was assessed to confirm cell death. To further validate apoptosis in NIH/3T3 cells induced by puromycin and to compare with PI-based results, Annexin V staining was subsequently performed. Annexin V detects early-stage apoptosis by targeting phosphatidylserine (PS), a phospholipid normally located inside the cell membrane. During apoptosis, membrane integrity is compromised, causing PS to translocate to the outside of the cell membrane and become exposed on the cell surface. In the presence of calcium ions, Annexin V specifically binds to this externalized PS, allowing the identification of apoptotic cells [45,46,47].

Similarly to PI staining analysis, cells were cultured on a 6 well plate at a density of 2 × 10^5^ cells/well to treat puromycin at a concentration of 6.67 μg/mL, and after drug treatment, cells were collected using cell scraper and washed with PBS. RNase (0.2 mg/mL) was added to the cell suspension for 30 min, and then 0.5 μg/mL Annexin V (BioLegend, San Diego, CA, USA) was added at room temperature for 15 min. The presence of apoptosis was detected by genetic cytometry using FACS Calibur (Becton-Dickinson, Franklin Lakes, NJ, USA).

## 3. Results

### 3.1. Measurement of Impedance Biosensor According to Frequency

**Figure 2:**
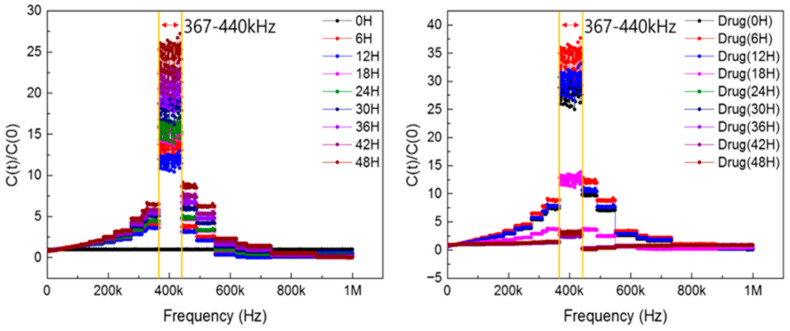
Identification of the most sensitive frequency range in impedance biosensors.

**Figure 3:**
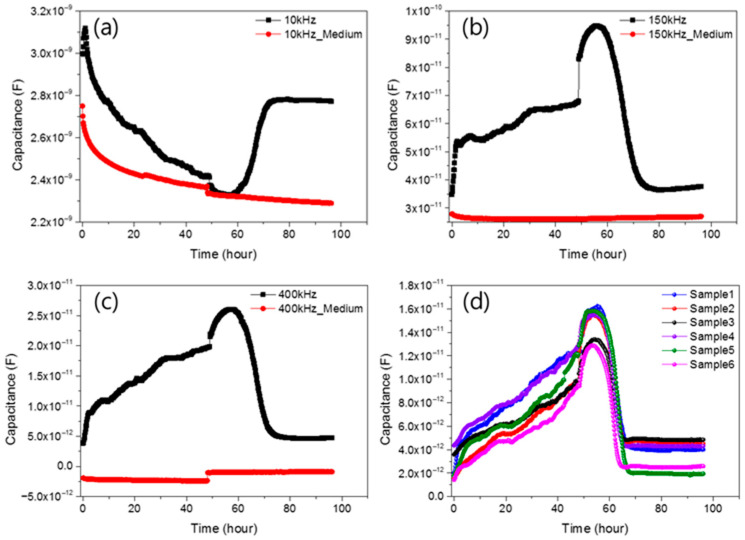
3 displays representative impedance profiles measured at (**a**) 10 kHz, (**b**) 150 kHz, (**c**) 400 kHz, and (**d**) reproducibility of six different impedance biosensor samples along with a control graph (red) representing a sensor with only medium (DMEM) and no cells.

To identify the optimal frequency range for measuring the impedance of cells cultured on the sensor, capacitance and conductance were recorded at 1 h intervals across a frequency sweep. These measurements were performed under both cell proliferation and apoptosis conditions to observe how cell related impedance signals varied over time. The results enabled the identification of the frequency range most sensitive to changes in capacitance associated with cell behavior. Figure 2 shows the frequency-dependent variation in C(t)/C(0) for a representative sample, confirming that the most responsive region lies between 367 kHz and 440 kHz. We specify that Figure 2 is a representative single-sensor frequency map derived from full sweeps, and we state that the identified sensitive band was reproduced across all six sensors (see Figure 3d and Section 2 Materials and Methods). Based on the overall analysis, the highest-sensitivity band was identified as 350–550 kHz. To strengthen this frequency identification, we repeated full sweeps on six independent sensors measured on different dates; in all cases, the identified bands overlapped within 350–550 kHz, with the aggregate mean clustering near ~400 kHz. Summary statistics for the bands and the overall mean are provided in Appendix A.7 Figure A7, and between-sensor overlays at 400 kHz are shown in Figure 3d.

### 3.2. Cell Growth and Drug Response in Impedance Biosensors with Time

In order to confirm the measurement result of the change in capacity according to the frequency sweep, the change in capacity and conductance over time was measured. Both the low and high frequency range were measured to further increase the reliability of the C(t)/C(0) frequency graph. The frequencies were 1 kHz, 3 kHz, 10 kHz, 50 kHz, 100 kHz, 150 kHz, 300 kHz, 400 kHz, 500 kHz, 600 kHz, 700 kHz, and 800 kHz, and measurements were performed in a total of 12 frequency. First, after seeding 10,000 cells in the well of the sensor, the cell growth was measured every 10 min for 48 h to observe the cell condition. After that, the death of cells grown by injecting drugs was measured and observed every 10 min. As a result, in the case of capacitance, the change in value was not stable up to 50 kHz, and it was confirmed that the normal graph shape was gradually found until the range of 100 kHz. It was confirmed that the shape of the graph appeared stably in a higher frequency range than after 300 kHz. Looking at the graph in Figure 3, the impedance graphs measured at (a) 10 kHz, (b) 150 kHz, (c) 400 kHz are shown along with the control graph (the impedance result measured only by the medium, red).

To verify reproducibility, measurements were repeated on six sensors, with results shown in Figure 3d at 400 kHz. While the extent of signal change varied slightly due to differences in cell growth and death, all samples followed the same overall trend. Capacitance values at initial seeding and after complete cell death were nearly identical across samples, confirming consistency.

To confirm that capacitance changes were due to cell growth, control sensors without cells were measured under identical conditions. These showed minimal variation during incubation and after drug treatment, indicating that observed changes originated from cellular activity. At 400 kHz, capacitance increased rapidly within the first 4 h after seeding, then gradually over the next 48 h. This reflects initial cell settling, followed by progressive growth and attachment on the sensor surface. Capacitance increased as cells proliferated over 48 h. After puromycin injection, a drug-induced response was monitored for an additional 48 h. Immediately after drug administration, a distinct capacitance shift was observed initially rising for approximately 9 h, then decreasing sharply. This transient increase is attributed to osmotic swelling from drug−cell interactions [48]. The subsequent drop in capacitance indicates cell death. To verify reproducibility, measurements were repeated on six sensors, with results shown in Figure 3d at 400 kHz. While the extent of signal change varied slightly due to differences in cell growth and death, all samples followed the same overall trend. Capacitance values at initial seeding and after complete cell death were nearly identical across samples, confirming consistency.

### 3.3. Process of Cell Growth Within the Impedance Biosensor Well

**Figure A6:**
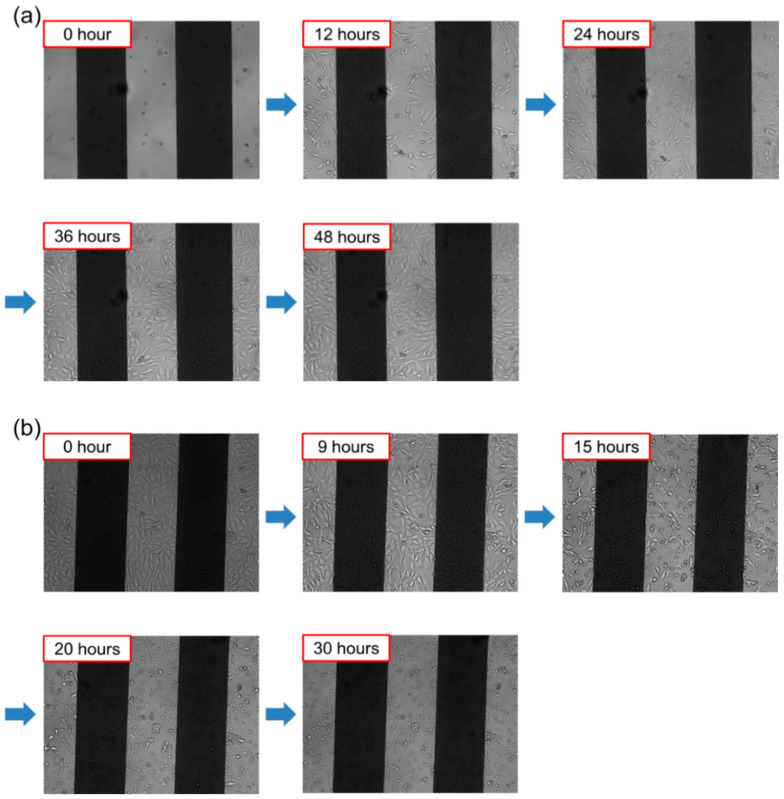
(**a**) The growth of cells and (**b**) the process of apoptosis of cells after drug treatment as observed under the JuLITM Br & FL microscope.

**Figure A7:**
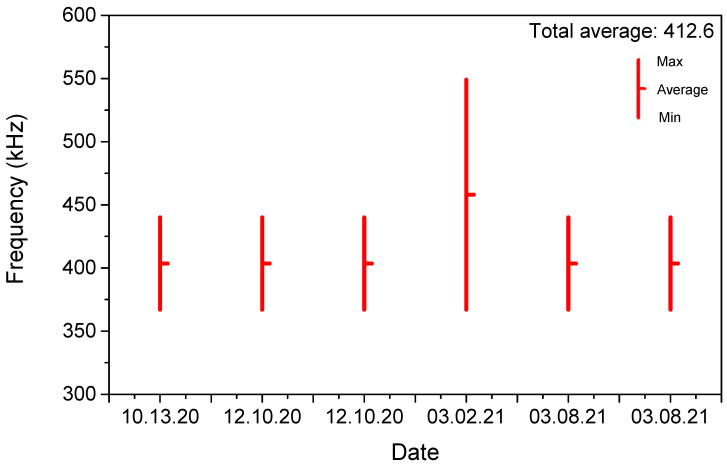
Sensitive frequency bands and their average frequencies according to frequency sweep results of several samples.

Figure A6a shows real time images captured by JuLI™ Br & FL during cell growth and death. Images taken every 12 h illustrate gradual cell proliferation, with the well reaching full confluence by 48 h. At this point, puromycin (6.67 μg/mL) was added. In Figure A6b, little change is observed during the first 9 h post-injection, but rapid cell death becomes evident between 9 and 24 h, as shown in 5 h interval images.

### 3.4. Intra-Sensor Cell Share in Images Observed by Microscope

**Figure 4:**
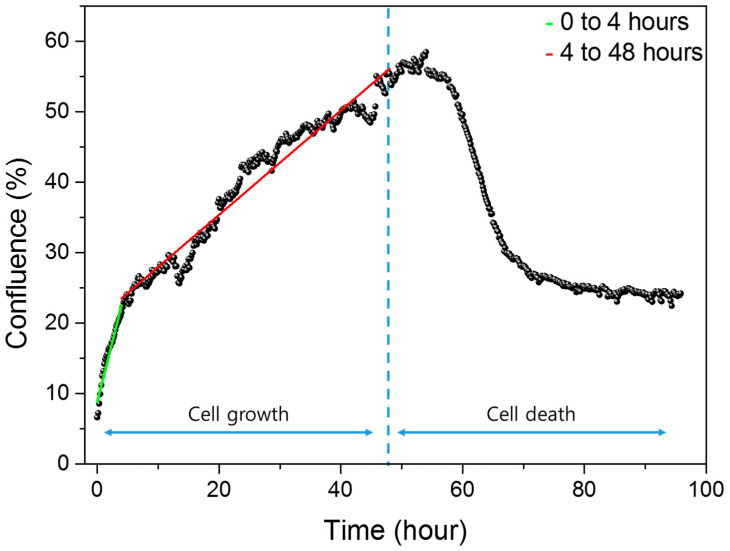
Confluence data quantifying the area occupied by cells in the electrodes in the well with microscopic images during cell growth and death.

Cell growth and death were analyzed using NanoEnTek’s JuLI™ Br & FL, which generated confluence graphs representing the percentage of area occupied by cells. As shown in Figure 4, confluence increased rapidly during the initial hours after seeding, reflecting the settling of cells to the bottom of the well. After about four hours, the confluence gradually increases as the cells completely sink and divide as they adhere to the floor. This shift in growth rate is evident from the change in slope before and after 4 h. To quantify this, curve fitting was performed separately for the periods of 0–4 h and 4–48 h. The resulting fitted curves are shown in green (early phase) and red (later phase), with the respective equations provided.

(1) $${\mathrm{Confluence}}_{0\text{–}4}\;(\%)=3.42918X+8.79963$$

(2) $${\mathrm{Confluence}}_{4\text{–}48}\;(\%)=0.7407X+20.537$$

In our previous study [49], we characterized the differences in cellular growth behavior between the early and late phases and demonstrated that these phase-specific behaviors were distinctly reflected in the impedance signals. These biological distinctions were consistently mirrored in the impedance measurements, with reproducible signal patterns corresponding to each growth stage. This correlation confirms that the phase-specific characteristics of cell behavior can be reliably captured through impedance measurements, thereby enhancing the interpretability and validity of image-based confluence analysis within our impedance biosensor platform.

The confluence graph from 0 to 4 h reflects cell morphology changes and settling behavior, as cells sink and attach to the sensor surface. From 4 to 48 h, the graph represents gradual cell proliferation.

Following 48 h of growth, puromycin was administered, and confluence decreased as apoptosis progressed. At 48 h, the confluence value was 55.39%, not approaching 100%, due to dark electrode regions in the image that hinder cell detection. Similarly, at the onset of cell death, the initial confluence was 53.98%. Minor fluctuations in the graph are attributed to image analysis variability caused by cell movement.

### 3.5. Biological Measurements According to Cell Growth and Drug Reaction

To compare electrical signals from impedance biosensors with biological data, cell growth and death were also evaluated using MTS assay and fluorescence-activated cell sorting (FACS). MTS assay was used to assess cell proliferation and viability, while FACS provided a clearer analysis of the apoptosis process.

#### 3.5.1. Cell Proliferation Analysis

**Figure A8:**
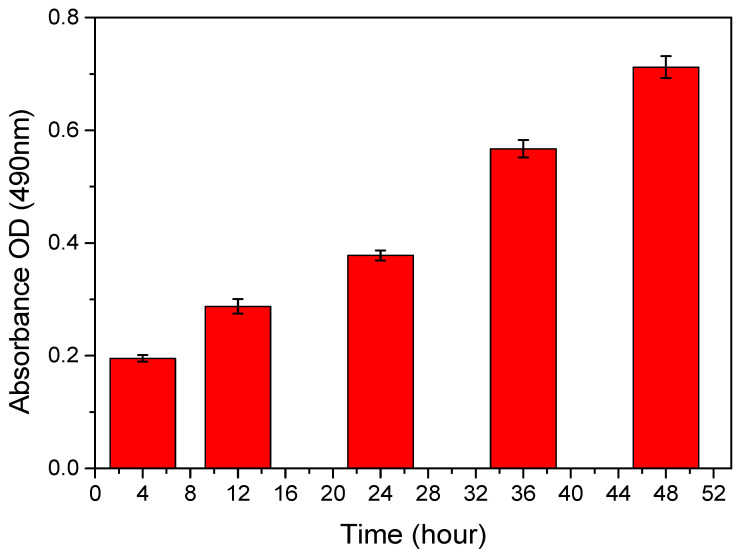
Cell growth MTS assay experimental results for 48 h.

Cell proliferation of NIH/3T3 cells was evaluated using the MTS assay, which measures the reduction in the tetrazolium compound MTS into formazan by mitochondrial enzymes in viable cells [50]. As shown in Figure A8, absorbance increased from 0.1877 to 0.7265 over time, indicating time-dependent cell growth. Compared to the initial value, cell proliferation increased by 1.44 times at 12 h, 1.96 times at 24 h, 2.95 times at 36 h, and 3.87 times at 48 h.

#### 3.5.2. Cell Death Analysis After Puromycin Treatment

Puromycin, a 3′-terminal analog of aminoacyl-tRNA, incorporates non-specifically into elongating polypeptide chains, leading to premature termination of translation and induction of apoptosis [37,51]. During apoptosis, endonuclease activation causes DNA fragmentation, which can be detected using intercalating dyes such as Propidium Iodide (PI) [2]. To assess the extent of apoptosis induced by puromycin, DNA content was analyzed via flow cytometry. At 0 h, NIH/3T3 cells displayed a typical cell cycle distribution with G1 (2n), S (2n–4n), and G2/M (4n) phases, and The area of sub-G1 (less than 2n) is only 0.47%, meaning that most cells are alive at 0 h. However, the sub-G1 population increased progressively, reaching 90.4% at 45 h after puromycin treatment.

Flow cytometry results for cell death are presented in Figure A8. Data were collected every 4 h up to 32 h, then at 6–7 h intervals as most cells had undergone apoptosis beyond that point. The figure highlights key transitions in cell death progression. Apoptotic cells, detected in the sub-G1 region (absorbance range: 0–180 on the *x*-axis), represent cells that have exited the normal cell cycle. From 0 to 8 h post-treatment, minimal changes were observed. A leftward shift in the graph began at 12 h, indicating early apoptosis. A significant increase in apoptotic cells occurred between 16 and 20 h, with a rapid shift continuing through 24 h.

### 3.6. Comparison of Impedance Biosensors and Biological Measurements

**Figure 5:**
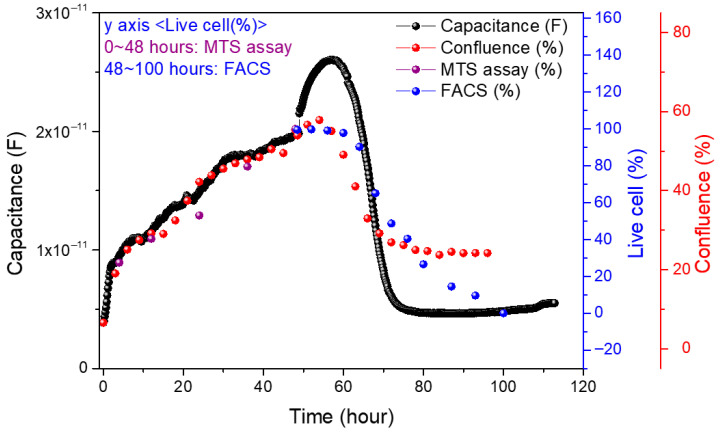
Comparison of impedance biosensor measured electrical signals with MTS assay and FACS data.

Figure 5 compares cell growth and death trends measured by impedance biosensors, confluence analysis, and MTS assay. The three datasets show highly consistent growth patterns, demonstrating the reliability of impedance-based measurements. MTS assay results begin at 4 h post-seeding due to the time required for cell attachment and growth. In contrast, impedance biosensors can detect cellular changes immediately after seeding, capturing capacitance increases from 0 to 4 h as cells settle and contact the sensor surface. This early sensitivity is also reflected in the confluence data, where the rate of increase is steeper in the initial hours. These findings indicate that impedance biosensors can sensitively detect subtle changes in cell behavior from the earliest stages of attachment and proliferation.

In the cell death phase, data from impedance biosensors, confluence analysis, and FACS were compared, as shown in Figure 5. Among the three, impedance biosensors demonstrated the highest sensitivity in detecting early apoptotic changes. Unlike confluence and FACS, which showed delayed responses, impedance signals captured cell state changes immediately following drug administration. While confluence values decreased to about half the level observed during growth, impedance measurements showed a complete return to baseline (0 h value) once all cells had died. This discrepancy arises because confluence measures the surface area occupied by cells, while impedance reflects both cell attachment and viability. Comparison with FACS further supports this finding. Microscopy and impedance data indicated near-complete cell death by 20 h and total death by 24 h post-injection. However, FACS still showed 49% viable cells at 24 h, suggesting that impedance biosensors detect apoptosis with greater sensitivity than optical staining-based methods.

**Figure A9:**
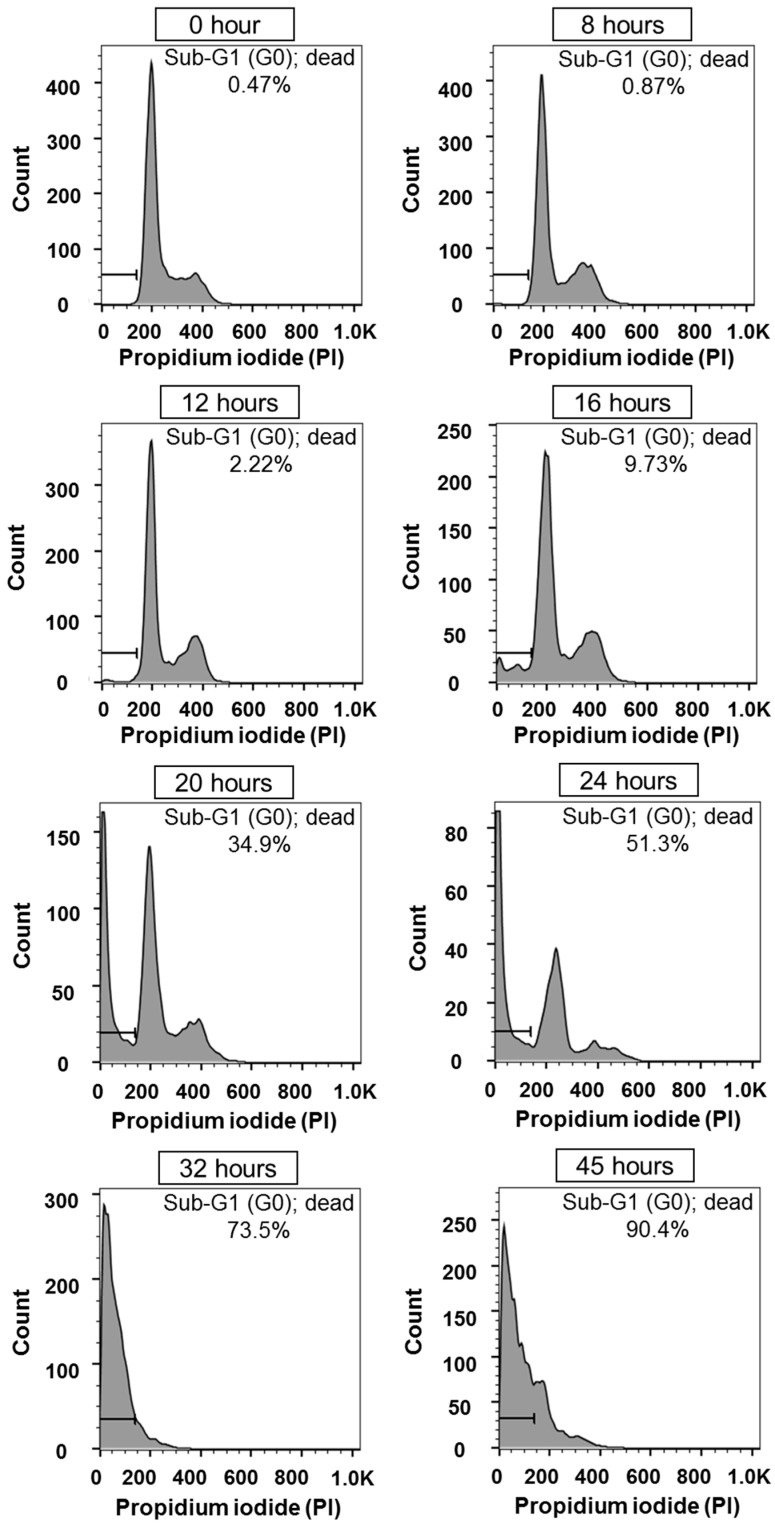
FACS measurement results using propidium iodide (PI) for 52 h after drug injection.

**Figure A10:**
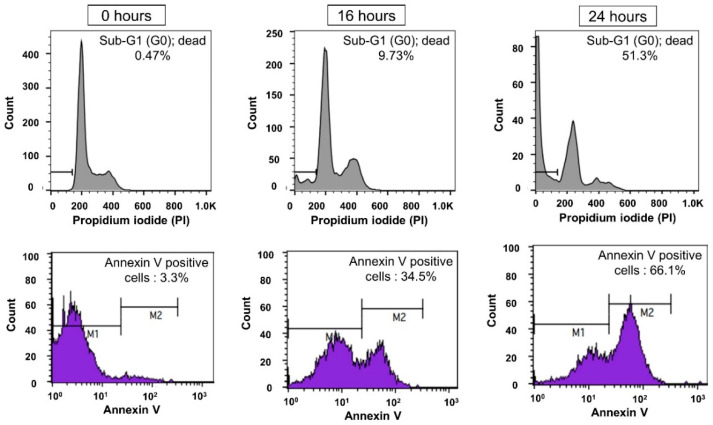
Comparison of FACS experimental results of PI and Annexin V.

FACS analysis using PI staining showed that 49% of NIH/3T3 cells remained viable 24 h after puromycin treatment, based on cell cycle profiles. To further validate these results, Annexin V staining was performed. As shown in Figure A9 and Figure A10, the PI-based sub-G1 analysis indicated 49% viability at 24 h, while Annexin V staining revealed 34% of cells still alive at the same time point. In contrast, microscopic images confirmed that nearly all cells had died and exhibited a shrunken morphology by 24 h. Similarly, impedance biosensor data reflected complete cell death. These findings suggest that impedance biosensors can detect apoptosis more sensitively and efficiently than conventional methods, enabling real-time monitoring of cell viability under live incubation conditions.

## 4. Discussion

This work establishes an impedance-based biosensing platform that captures the full timeline of NIH/3T3 growth and drug-induced apoptosis in a label-free, non-destructive, and continuous manner. Three results are central. First, a broad sweep (1 kHz–1 MHz) revealed a high-sensitivity band centered at ~367–440 kHz (overall 350–550 kHz). Second, time-lapse measurements at 12 discrete frequencies confirmed that the low-frequency response (≤10 kHz) is governed by electrode polarization and bulk medium conductivity, so cell-dependent variations are largely masked; cell-specific features emerge near 100–150 kHz and trajectories become stable and reproducible at ≥200 kHz. This trend is consistent with ECIS/electrochemical-impedance theory, in which low-f behavior is interfacial-dominated whereas mid–high-f signals (β-dispersion) report cell–substrate coupling and membrane capacitance [52,53]. Third, at a representative 400 kHz, capacitance captured the entire biological sequence—rapid rise over the first 4 h, gradual increase during 48 h, a transient post-drug rise (~9 h), and a sharp decline to baseline within ~24 h—providing an interpretable electrical fingerprint of apoptosis-driven detachment.

A key methodological decision was to emphasize the capacitance component rather than total impedance or resistance alone. Capacitance is directly modulated by membrane permittivity, cell–substrate sealing, spreading area, and junctional reorganization, making it particularly responsive to early morphological/physiological changes that precede overt death. The transient increase after puromycin—a feature that could be averaged out in scalar impedance—was consistently observed and is plausibly explained by osmotic swelling and adhesion changes during the early drug–cell interaction phase. Component-level interpretation thus improves both sensitivity and biological specificity of ECIS-type readouts.

Orthogonal measurements support these interpretations. Medium-only controls exhibited minimal drift during incubation and after drug addition, indicating that the observed electrical changes originate from cellular activity rather than bath artifacts. Microscopy-based confluence tracked the same phases: a steeper early slope (0–4 h; settling/adhesion) followed by slower growth (4–48 h) and a decline after drug exposure. Small offsets in absolute confluence (e.g., <100% at 48 h) are attributable to dark electrode regions in the field of view; this explains why confluence may plateau above zero even when impedance has returned to its baseline. Biological assays further contextualized the timeline. MTS reported monotonic proliferation consistent with capacitance increases during growth. During the death phase, flow cytometry revealed a delayed trajectory relative to impedance and imaging: PI-based sub-G1 analysis indicated ~49% viability at 24 h, while Annexin V staining indicated ~34% viability at the same time. In contrast, impedance and microscopy had already reached near-complete death by ~20–24 h. This divergence is reasonable: PI/Annexin V reflect intracellular DNA content or externalized phosphatidylserine under fixed assay conditions, whereas impedance responds immediately to loss of adhesion and sealing, enabling earlier detection of apoptotic progression under live incubation.

The frequency dependence observed here is also informative. Below ~50 kHz, electrode polarization, double-layer capacitance, and stray impedances reduce biological contrast, explaining the instability and poorer reproducibility in this band. Above ~300 kHz, the current paths become increasingly sensitive to cell coverage and membrane capacitive effects, yielding stable, sample-to-sample-consistent trajectories. Identifying a narrow operational window (e.g., 367–440 kHz; representative 400 kHz) thus streamlines acquisition, facilitates cross-study comparison, and enables practical deployment where a single frequency (or a small set) is preferred.

This study has limitations. It focuses on one cell type (NIH/3T3) and one cytotoxic compound (puromycin). Extending to additional cell lines (epithelial, immune, cancer) and drug classes (targeted agents, anti-inflammatory compounds, membrane-active antibiotics) will test generalizability. The observation window (96 h) captures acute cytotoxicity; longer monitoring could uncover delayed or adaptive responses. Finally, while we used imaging and standard biochemical assays for validation, integrating molecular readouts (e.g., caspase activity, or transcriptomics) would deepen mechanistic interpretation of the transient and decline phases.

In sum, by pinpointing an optimal operating band and leveraging capacitance-centric analysis, this work shows that impedance biosensing can detect drug-induced apoptosis earlier and with higher temporal fidelity than conventional endpoint assays, while remaining label-free and non-destructive. The approach is well-suited for quantitative screening, cytotoxicity profiling, and longitudinal monitoring, and it provides a tractable foundation for multimodal workflows that couple real-time electrical sensing with high-content imaging and molecular readouts.

## 5. Conclusions

In this study, an impedance biosensor capable of measuring cell conditions was manufactured and conditions for cell growth in the sensor were set. Cell growth and death were observed through the manufactured sensor. At this time, frequency sweeps were performed to identify areas that sensitively capture cell state changes according to frequencies. As a result, it was confirmed that the signal was the most sensitive between 367 and 440 kHz. Therefore, in order to determine how the cell state appears from low frequencies to high frequencies, a total of 12 frequencies were targeted, namely 1 kHz, 3 kHz, 10 kHz, 50 kHz, 100 kHz, 150 kHz, 300 kHz, 400 kHz, 500 kHz, 600 kHz, 700 kHz, and 800 kHz, and the results were measured. NIH/3T3 cells were grown in a CO_2_ incubator for 48 h and then puromycin was injected at a concentration of 6.67 μg/mL and observed for 48 h. It was confirmed that almost all cells died after 24 h of drug injection. As a result of measuring the cell state through an impedance biosensor, it was confirmed that the electrical signal had a greater influence on the medium than the cell state in the low frequency range of 1–10 kHz. From the 100–150 kHz area, it was confirmed that the electrical signal of the impedance biosensor reflected the cell state, and from 300 kHz to 800 kHz, the sensor signal stably appeared. At this time, the impedance biosensor electrical signals at 400 kHz corresponding to the most sensitive frequency range obtained as a result of the experiment were verified and compared with various biological analysis methods such as MTS assay, FACS, and microscopic image confluence. As a result, it was confirmed that cell measurement with impedance biosensors can be monitored more easily, efficiently, and sensitively compared to conventional biological analysis methods. Therefore, the impedance biosensor manufactured in this study shows the possibility to be used as a cell growth and drug reaction monitoring method.

## Figures and Tables

**Table 1:** Representative impedance-based studies on adherent cell cultures and comparison with this work.

Study	System and Substrate	Cell Line	Application and Readout	Key Points	Limitation vs. This Work
ref.1 [7]	xCELLigence RTCA system	Fibroblasts	Cytotoxicity of Ga/In liquid metal alloys	Applies RTCA impedance to novel liquid-metal biomaterials.	limited quantitative metrics; no parallel biological assays.
ref.2 [8]	ECIS with small gold electrodes	L929, CT-26	Cytotoxicity of fungal extracts (Z, R, C)	Time-resolved Z/R/C for dose-dependent extract toxicity.	Fixed ECIS frequencies; no frequency-sweep optimization
ref.3 [9]	Four-electrode impedimetric biosensor	HeLa	Tamoxifen cytotoxicity (impedance)	Novel four-electrode design for label-free drug cytotoxicity.	limited frequency analysis; no multi-assay validation
ref.4 [10]	ECIS with small gold electrodes	GT1-7 and other cells	Cytotoxicity of natural product extracts	Resolves complex time-dependent cytotoxic responses	No frequency-sweep optimization; no parallel viability/apoptosis assays
ref.5 [11]	ECIS-based epithelial barrier biosensor	Intestinal epithelial cells	Barrier integrity and damage classification	Quantitative barrier function parameters	Barrier-focused; no fibroblast drug cytotoxicity
This work	Platinum impedance biosensor on slide glass + PDMS well	NIH/3T3	Real-time growth and puromycin apoptosis MTS/FACS/Annexin V comparison	Frequency-optimized sensing with quantitative drug metrics and three-assay validation	Provides integrated, quantitative validation of impedance biosensing as an alternative to conventional assays

## Data Availability

The original contributions presented in this study are included in the article. Further inquiries can be directed to the corresponding author.
